# Draft Genomes of Methanocalculus taiwanensis P2F9704a^T^ and Methanocalculus chunghsingensis K1F9705b^T^, Hydrogenotrophic Methanogens Belonging to the Family *Methanocalculaceae*

**DOI:** 10.1128/mra.00792-22

**Published:** 2022-09-06

**Authors:** Sheng-Chung Chen, Shu-Jung Lai, Yi-Ting You, Chao-Jen Shih, Yen-Chi Wu, Chih-Hung Wu, Ching-Hua Liao

**Affiliations:** a School of Chemistry and Chemical Engineering, Hubei Polytechnic University, Huangshi City, Hubei, People’s Republic of China; b School of Resources and Chemical Engineering, Sanming University, Sanming City, Fujian, People’s Republic of China; c Department of Life Sciences, National Chung Hsing University, Taichung City, Taiwan, People’s Republic of China; d Graduate Institute of Biomedical Sciences, China Medical University, Taichung City, Taiwan, People’s Republic of China; e Research Center for Cancer Biology, China Medical University, Taichung City, Taiwan, People’s Republic of China; f Bioresource Collection and Research Center, Food Industry Research and Development Institute, Hsinchu, Taiwan, People’s Republic of China; g College of Environment & Safety Engineering, Fuzhou University, Fuzhou City, Fujian, People’s Republic of China; Portland State University

## Abstract

The family *Methanocalculaceae* comprises hydrogen- and formate-utilizing methanogens. Here, we report two additional draft genome sequences of *Methanocalculaceae*, those of Methanocalculus taiwanensis P2F9704a^T^ (equivalent to BCRC 16182^T^ and DSM 14663^T^) and Methanocalculus chunghsingensis K1F9705b^T^ (equivalent to DSM 14646^T^ and OCM 772^T^), which were selected for further species delineation and comparative genomic analyses.

## ANNOUNCEMENT

The members of the genus *Methanocalculus* belong to the *Methanocalculaceae* family. *Methanocalculus* spp. use only formate and hydrogen as electron donors for methanogenesis and require acetate as a carbon source (1). To date (August 2022), the genus *Methanocalculus* comprises six species with validly published names: Methanocalculus halotolerans (2), Methanocalculus pumilus (3), Methanocalculus taiwanensis (4), Methanocalculus chunghsingensis (5), Methanocalculus natronophilus (6), and Methanocalculus alkaliphilus (7). *Methanocalculus* isolates inhabit a wide range of environments, such as an estuary, a marine water aquaculture fishpond, the leachate of a sea-based site for solid waste disposal, a saline oil reservoir, soda lakes, and anaerobic digesters (2–9). Here, we report two draft genomes of M. taiwanensis P2F9704a^T^ and M. chunghsingensis K1F9705b^T^ that provide further understanding about species delineation and microbial adaptation to various environments.

Strains P2F9704a^T^ and K1F9705b^T^ were isolated from an estuary of Erlin River and a marine water aquaculture fishpond near Wong-Gong, Taiwan, respectively (4, 5). Both strains were grown in anaerobic MB/W medium with 100 mM sodium formate and 5 mM sodium acetate and incubated at 37°C, according to methods described previously (10–12). Genomic DNA of each strain was isolated and purified using a modification of the methods of Johnson (13) and of Jarrell et al. (14). Briefly, cells of a 500-mL culture were collected and then lysed with sodium dodecyl sulfate (1% [wt/vol]). After phenol-chloroform extraction and isopropanol precipitation, the quantity and quality of the extracted DNA samples were checked by UV-visible spectrophotometry.

The genomes of both strains were sequenced at the Genomics BioSci & Tech Co., Ltd. (Taiwan) using the MiSeq platform (Illumina). For both strains, genomic DNA was sheared randomly, and a paired-end DNA library of 300 bp was constructed by using the TruSeq Nano DNA HT library prep kit and TruSeq DNA with 96 CD indexes (Illumina). The constructed library was sequenced using a MiSeq reagent kit v3 (600 cycles), and 6,537,788 and 4,735,720 reads were generated for strains P2F9704a^T^ and K1F9705b^T^, respectively. All generated reads were quality trimmed to obtain high-quality reads using Trimmomatic v0.36 (15). For the genome *de novo* assembly, the genome assembler of Shovill v1.0.4 (16) was applied. The sequencing protocol generated ~425-fold and ~547-fold mean coverage for the genomes of strains P2F9704a^T^ and K1F9705b^T^, respectively. Genes of the genome were identified using the Prokaryotic Genome Annotation pipeline (PGAP) of the National Center for Biotechnology Information (http://www.ncbi.nlm.nih.gov) (17). For the draft genome of strain P2F9704a^T^, a total of 69 contigs were obtained with an *N*_50_ value of 72,685 bp and a total size of 2,342,634 bp with a 50.41% GC content. The genome was predicted to harbor 2,348 genes. For strain K1F9705b^T^, a total of 51 contigs were obtained with an *N*_50_ value of 84,108 bp and total size of 2,134,545 bp with a 52.22% GC content. The genome was predicted to harbor 2,253 genes. No plasmid was identified in either genome using PlasmidFinder (18). Default parameters were used for all software unless otherwise specified.

### Data availability.

These whole-genome shotgun projects for strains P2F9704a^T^ and K1F9705b^T^ have been deposited in GenBank under the accession numbers VOTZ00000000 and JWHL00000000, respectively. The versions for each genome described in this paper are the respective first versions. The BioProject accession numbers for strains P2F9704a^T^ and K1F9705b^T^ are PRJNA559048 and PRJNA224116, respectively. The raw sequence reads for strains P2F9704a^T^ and K1F9705b^T^ have been deposited in the Sequence Read Archive under accession numbers SRR18085627 and SRR20645508, respectively.
